# Infants exploit vowels to label objects and actions from continuous audiovisual stimuli

**DOI:** 10.1038/s41598-021-90326-z

**Published:** 2021-05-26

**Authors:** Cristina Jara, Cristóbal Moënne-Loccoz, Marcela Peña

**Affiliations:** 1 Laboratorio de Neurociencias Cognitivas, Escuela de Psicología, Pontificia Universidad Católica de Chie, P.O. 7820436, Santiago de Chile, Chile; 2 Departamento de Ciencias de la Salud, Facultad de Medicina, Pontificia Universidad Católica de Chile, P.O. 7820436, Santiago de Chile, Chile

**Keywords:** Psychology, Paediatric research

## Abstract

Before the 6-months of age, infants succeed to learn words associated with objects and actions when the words are presented isolated or embedded in short utterances. It remains unclear whether such type of learning occurs from fluent audiovisual stimuli, although in natural environments the fluent audiovisual contexts are the default. In 4 experiments, we evaluated if 8-month-old infants could learn word-action and word-object associations from fluent audiovisual streams when the words conveyed either vowel or consonant harmony, two phonological cues that benefit word learning near 6 and 12 months of age, respectively. We found that infants learned both types of words, but only when the words contained vowel harmony. Because object- and action-words have been conceived as rudimentary representations of nouns and verbs, our results suggest that vowels contribute to shape the initial steps of the learning of lexical categories in preverbal infants.

## Introduction

Across cultures, sighted and hearing infants must discover word-visual referent associations from fluent contexts. For instance, verbal expressions such as “Look! the dog jumps” are usually associated with pointing gestures to a visual scene composed of many possible referents. The mechanisms underpinning the early steps of such learning remain poorly understood^1^.

Previous studies have shown that when exposed to continuous stimuli, 8-months-old infants can exploit the distributional cues between syllables to find words^2^ and between geometric figures to extract visual chunks^3^. In contrast, at the same age, infants fail to learn word-object associations from fluent audiovisual streams, even when the streams comprise similar stimuli^4^. Indeed, although 8-month-olds succeed to learn the words, they failed to associate them with the geometric figures presented synchronically. The author interpreted the results as an infants' bias to process speech over visual stimuli at this age. These negative results differ from others showing that infants learn new word-image associations when they take place in segmented instead of fluent contexts. For instance, after the exposure to a series of word-image associations presented isolated or embedded in short utterances, infants as young as 4-month-olds succeed in generalizing familiar nouns such as mommy and daddy^5^ and feet and hands^6^ to different visual referents, and familiar verbs such as “hit” to the corresponding gesture^7^. Infant brain data also confirm that 3-month-olds can learn word-object associations previously unseen after a brief exposure^8^ and 9-month-olds generalize new labels to novel exemplars of the object category^9^.

The learning of word-object and word-action associations in preverbal infants has been conceived as a rudimentary ability to represent words as lexical categories. While object-words are used for nouns, the action-words are for verbs^10^. Therefore, exploring the ability to match words with different types of visual referents can contribute to advance into a better understanding of the early steps of word learning development.

Here we explored whether 8-month-olds could learn object- and action-words after a brief exposure to fluent audiovisual streams, when the words conveyed either vowel or consonant harmony. Vowel and consonant harmony are phonological properties whereby vowels or consonants within a word systematically exhibit similarity in some aspects of the way they are pronounced. Previous studies showed that infants younger than 6 months exploit more vowels than consonants to learn new words from non-fluent contexts^11–13^. However, near their first birthday, infants switch this asymmetry and exploit mainly the consonants to succeed in the same task^12^. Additionally, a recent study showed that vowel harmony is better than consonant harmony at leading continuous speech segmentation in 7-month-old^14^.

To increase the engagement with the task, instead of geometric figures, the visual referents of our audiovisual streams were pictures of human faces or videos of human head gestures. Faces can be conceived as objects, even special ones but objects, because face and object processing relies on similar recognition methods such as feature extraction and pattern recognition^15^. The special value of faces would be related to the information they convey, which has social and semantic value^16^. Indeed, faces are salient from birth^17,18^ and facilitate mental computations that otherwise would not necessarily take place, such as individuation, tracking, and counting objects^19^. We expected that infants would perceive the words associated with pictures as object-words and those associated with videos as action-words. As in previous studies, we presented the words and visual referents in perfect temporal synchrony, because it has been reported as beneficial for word learning in non-fluent contexts^20^.

We hypothesized that when exposed to an audiovisual stream containing phonological and engaging cues, infants would learn object-words and action-words. Moreover, given the advantage of the vocalic harmony over the consonantal one to discover words from fluent speech^14^, we predicted that vowel harmony would benefit more than consonant harmony in the learning of object- and action-words.

**Figure 1:**
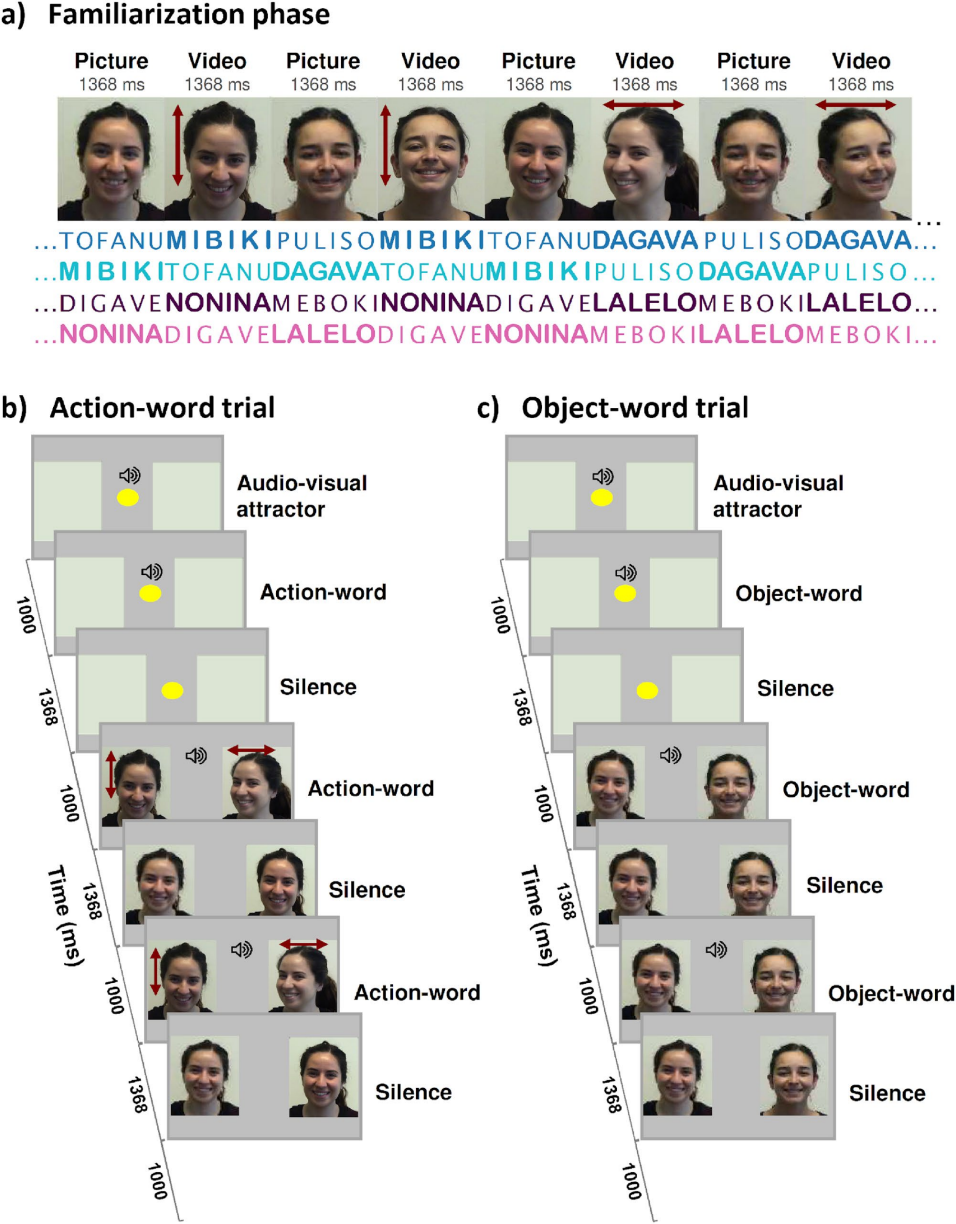
Experimental protocol. In (**a**), we illustrate the pictures and videos used in the familiarization phase in all experiments. In the videos, the vertical and horizontal arrows indicate up-and-down and left-to-right head gestures, respectively. The words used in each experiment are written below the images, in dark blue for Experiment 1, in cyan for Experiment 2, in dark purple for Experiment 3, and in magenta for Experiment 4. The words in bold were cued either with vocalic or consonantal harmony and were tested at the end of the familiarization phase. In (**b**) and (**c**), we show the structure of the test trials evaluating the learning of the object- and action-words, respectively. Each trial began with a central audiovisual attractor, followed by the auditory presentation of a word simultaneously displayed with two empty squares, one at each side of the screen. Then, the same word was repeated twice, separated by 1 s of silence, and synchronically displayed with the presentation of 2 head gesture’s videos, one by each side in (**b**) or with 2 face’s pictures, one per side in (**c**). We recorded the infants' visual behavior by using the eye-tracking technique.

To test our hypotheses, we ran four experiments. Each one evaluated a different group of healthy 8-months-old infants. We first familiarized the infants with one of three possible versions of a 2.1 min-long continuous audiovisual stream (Fig. 1a and Table S1), and immediately after, we evaluated the word-image association learning in 16 test trials.

The audiovisual streams contained four trisyllabic nonsense words, played monotonously. Each one appeared synchronically with the display of a picture or a video (Supplemental Video 1–4). Two words co-occurred with the presentation of the picture of two different women, one per woman. All pictures included the face, head, and shoulders. The other two words concurrently displayed with videos, one video showed an up-and-down head gesture and the other a left-to-right head gesture. Head gestures were similar to those used for negation and affirmation in many cultures. Both were made by the woman presented in the precedent picture.

We cared that the words of the audiovisual stream had a transitional probability (TP) between adjacent syllables equal to 1 within words and 0.5 between words, to facilitate the continuous speech segmentation into trisyllables. The visual track of the audiovisual stream was built by concatenating six images: 2 pictures of the faces of two different women, one per woman, 2 videos of an up-and-down head gesture, each one made by each woman, and 2 videos of a left-to-right head gesture, each one made per each woman.

The TP between any adjacent image was equal to 0.5. Because the pictures and videos alternated, we provided an extra signal for audiovisual stream segmentation. We called object-words to the trisyllables that co-occurred with the women's faces pictures and action-words to those that synchronically displayed with a head gesture made by any of the two women.

To avoid overloading the infants’ cognitive demands, in all experiments, we added the phonological cue only to 2 of the 4 words, and we tested only those words during the test phase. The vowel harmony comprised the repetition of the same vowel in the three syllables of the words, and the consonant harmony the repetition of the same consonant. After the familiarization, we evaluated the learning by using a visual preference procedure. In each trial, the infants simultaneously heard a word and saw two lateralized images. Only one of the two image co-occurred with the current word in familiarization (correct image) while the other image did not (incorrect image). We measured the accuracy in recognizing the word-image association by computing the proportion of the time that the infants' gaze fell over the correct and incorrect image. In experiments 1 and 3, we measured the learning of action-words by presenting each action-word simultaneously to the two head gestures, one over each side, both made by the same woman (Fig. 1b). Likewise, in experiments 2 and 4, we tested the learning of object-words by presenting each object-word simultaneously with the displaying of the pictures of the two women (Fig. 1c).

We expected that, if infants learned the word-image associations during familiarization, in the test phase they would look first and/or longer into the correct image.

## Results

### Test variables and data analysis

Previous infant’s eye-tracking studies report the analysis of the first gaze (FG), total looking time (TLT), and longest fixation (LF) to measure the infant’s preference and to compute accuracy^21^. The FG would better reflect orientation, while the TLT and LF would reveal sustained attention, searching behaviors^22^ and memory recognition^23^. We thus computed and analyzed those variables in each experiment.

We computed the percentage of the time that each infant watched the visual stimuli during the familiarization phase (TLT-pct). This measure informed us about the infants' engagement with the task during the phase when we expected that word-image associations were learned. In the test phase, we analyzed only the valid trials defined as the trials in which the infants' gaze was at the center of the screen just before the onset of the presentation of the lateralized images.

**Figure 2:**
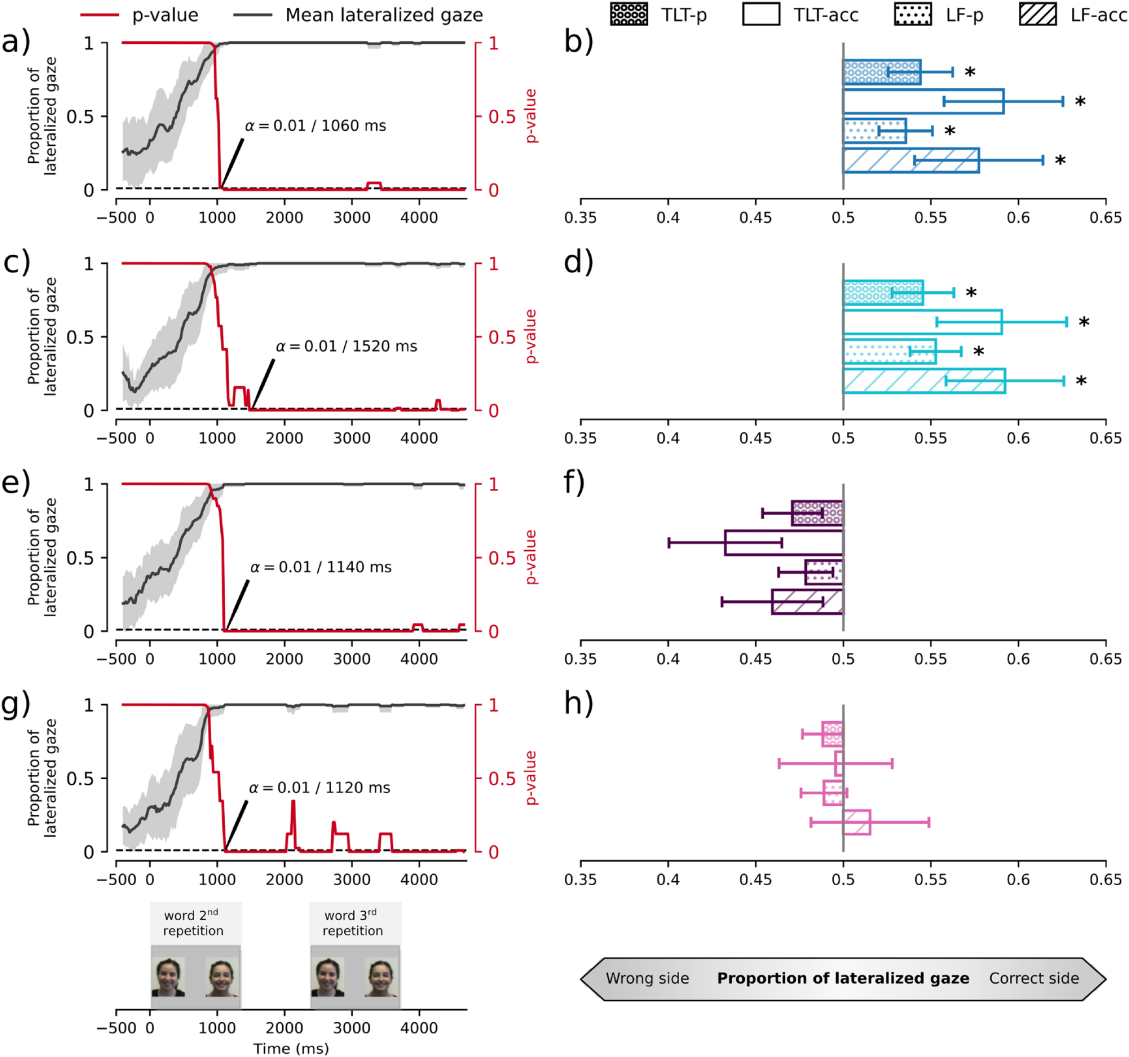
Window time for the analysis in each experiment. Left panels illustrate the time window when the mean gaze computed across all infants remained lateralized in (**a**) for Experiment 1, (**c**) for Experiment 2, (**e**) for Experiment 3, and (**g**) for Experiment 4. The black lines plot the proportion of lateralized gazes computed across all infants, from 500 ms before the presentation of the lateralized images to the end of the trial. The shadow indicates the standard deviation. The trial events inside the analysis window appear below the left panels. The y-axis encompasses from 0, when the gaze fell into the center of the screen, to 1 when was lateralized regardless of the side and correctness. The red lines depict the *P* value at each point of the same time window and show the period when the infants' gaze shifted from the center to the lateralized images and remained there (see Methods). The horizontal dotted line indicates *P* value < 0.01. The gray arrows indicate the onset of the time window we used for the analysis, which extended to 4700 ms in all experiments. The right panels show the accuracy for Experiment 1 in (**b**), Experiment 2 in (**d**), Experiment 3 in (**f**), and Experiment 4 in (**h**), illustrated as the mean TLT-p, TLT-acc, LF-p, and LF-acc. In the x-axis, 0.5 indicates no preference or mean gaze at chance level, values greater than 0.5 indicate a visual preference towards the correct image, while those below 0.5 a preference towards the incorrect image. The asterisks indicate a significant difference from the chance at *P* < 0.05.

We applied a data-driven approach over the valid trials to identify a temporal window when we must test our hypothesis (see “Methods”). Specifically, we identified a time window when, in the valid trials, all infants switched their gaze from the center to the lateralized images, regardless of the side and the correctness of the response (Fig. 2, left panels). This procedure allowed us to consider in our analysis the intra- and inter-subject variability in latency and duration of the visual behavior previously reported in eye-tracking infant studies^21^.

After identifying the temporal window, in each valid trial of each infant, we computed the FG, the TLT, and LF for correct and incorrect images over the mentioned window. The FG was assigned as 1 when it fell into the correct image and 0 otherwise. The TLT corresponded to the cumulative sum of time directed to the correct and incorrect images. The TLT proportion (TLT-p) for the correct image was computed by dividing the TLT over the correct image by the sum of TLT toward the correct and the incorrect images. Additionally, to obtain a discrete measure of accuracy, we transformed the TLT-p into a binary value by assigning 1 every time that the TLT-p was greater for the correct than for the incorrect image and 0 otherwise (TLT-acc). With a similar procedure, the LF was also analyzed as a proportion (LF-p) and accuracy (LF-acc). The LF-p was computed by dividing the LF over the correct image by the sum of LF over the correct and incorrect images. The LF-acc was transformed to 1 when the LF-p was greater for the correct than for the incorrect image and 0 otherwise. Finally, we submitted the above-described variables to statistical analysis, using *t*-test and Chi-square for comparisons inside each experiment and ANOVA for comparisons between experiments. All variables had normal distribution (Table S2) and we applied multiple comparison corrections when necessary.

### Experiment 1

In this experiment, we measured the learning of the action-words, which we cued by using vowel harmony (see Video S1). We evaluated 33 healthy infants, where 10 were excluded from the analysis because 6 presented fussiness and 4 contributed with less than 5 valid trials, remaining 23 infants for the analysis (11 females, mean age = 7.961 ± 0.445 months, see Table S3 & Table S4 for detailed bio-demographic data).

#### Familiarization

During familiarization the infants looked at the screen 64.6 ± 16.2% of the full duration of the stream, confirming they had the opportunity to learn. A post-hoc analysis showed the infants explored the eyes of the images (93.6% of the time), with only 13.1% of the time exploring the mouth.

#### Test

We evaluated the learning of the action-words in 16 trials, 8 per word, and the infants contributed on average with 8.7 ± 3.1 valid trials each (range = 5 to 15).

The time window when the group of infants maintained their gaze over the lateralized images was from 1060 to 4700 ms after the onset of the audiovisual presentation (at *P* < 0.01 Fig. 2a). The 1060 ms corresponded to the last syllable of the second presentation of the word. In this time window, the infants’ gaze remained watching the regions of interest we expected, i.e., left, center, and right areas (Figure S1).

As predicted, we found that in average, the infants looked significantly longer over the correct than the incorrect video (mean TLT = 1568 ± 294 ms and 1316 ± 361 ms respectively, *t*_(22)_ = 2,314, *P* = 0.030, Cohen’s d = 0.720). Moreover, the mean TLT-p, TLT-acc, LF-p and LF-acc were significantly greater than chance (*t*_(22)_ = 2.389, *P* = 0.026, Cohen’s d = 0.500; *t*_(22)_ = 2.694, *P* = 0.013, Cohen’s d = 0.562; *t*_(22)_ = 2.336, *P* = 0.029, Cohen’s d = 0.487; and *t*_(22)_ = 2.111, *P* = 0.046, Cohen’s d = 0.441, respectively) (Fig. 2b), confirming that the infants looked longer to the correct video. We did not find significant differences in the first gaze for correct and incorrect videos (*t*_(22)_ = 0.962, *P* = 0.347, Cohen’s d = 0.200).

Although women, actions, words, and word-action pairs were counterbalanced across infants (Table S1), we explored the possibility that infants may have preferred particular items. We did not find any significant effect in any comparison (at *P* > 0.920, Table S5). Additionally, we did not find significant correlations between any visual variable and the time exploring the stream during familiarization (*P* > 0.1) or the number of valid trials (*P* > 0.1), rejecting the possibility that our results related to the interest to explore the visual stimuli. Finally, we did not find that infants learned in the test phase (see Figure S2).

Together, our results indicated that when the action-words of the audiovisual streams conveyed vowel harmony, the infants succeeded in learning the word-action associations made by different women. To some extent, infants generalized the word across individuals. With this result, we proceeded to test if vowel harmony also helped the discovery of object-words from fluent audiovisual streams.

### Experiment 2

Here we added vowel harmony to the object-words only (see Video S2). We evaluated 37 healthy infants, 16 of them were excluded from the analysis, 6 because of fussiness and 10 because they had less than 5 valid trials, remaining 21 infants for further analysis (11 females, mean age = 7.964 ± 0.238 months, see Table S3 and Table S4).

#### Familiarization

The TLT-pct during familiarization was 68.2 ± 25.7%, indicating that infants were engaged with the task and had the opportunity to learn. Similar to Experiment 1, the infants’ mostly looked over the eyes area of the pictures (91.5%).

#### Test

The infants contributed on average with 9.2 ± 2.8 valid trials each (range = 5 to 16). The time window for the lateralized gaze analysis was from 1520 to 4700 ms (at *P* < 0.01, Fig. 2c), where 1520 ms corresponded to the silence after the second presentation of the word. As in Experiment 1, the infants’ gaze mostly fell into the expected regions of interest (Figure S1).

Similar to Experiment 1, we found that the infants looked significantly longer over the correct than the incorrect picture (mean TLT = 1380 ± 276 ms and 1165 ± 300 ms respectively, *t*_(20)_ = 2,437, *P* = 0.025, Cohen’s d = 0.493). The Fig. 2d illustrates the results of the mean TLT-p, TLT-acc, LF-p and LF-acc for this experiment. Moreover, all gaze variables were significantly greater than chance (*t*_(20)_ = 2.578, *P* = 0.018, Cohen’s d = 0.537 for TLT-p; *t*_(20)_ = 2.440, *P* = 0.024, Cohen’s d = 0.532 for TLT-acc; *t*_(*20*)_ = 3.607, *P* = 0.002, Cohen’s d = 0.787 for LF-p; and *t*_(*20*)_ = 2.743, *P* = 0.013, Cohen’s d = 0.598 for LF-acc). Similar to Experiment 1, FG was not different to chance (*t*_(20)_ = − 1.820, *P* = 0.071, Cohen’s d = − 0.397).

We neither find any bias to select particular items (*P* > 0.368), nor significant correlations between any visual variable and the time exploring the screen during familiarization (*P* > 0.3) or the number of the valid trials (*P* > 0.1). Infants did not learn during the test phase (see Figure S2).

The results of Experiment 2 replicated the results of Experiment 1, but now showing that vowel harmony facilitates the learning of word-object associations from continuous audiovisual streams. We then proceeded to evaluate whether consonant harmony facilitated word-image learning from audiovisual streams.

### Experiment 3

Experiment 3 mirrored Experiment 1, but now we added consonant harmony instead of vowel harmony to the action-words (see Video S3). We evaluated 26 healthy infants but 6 were excluded from the analysis, 3 because of fussiness, and 3 because they had less than 5 valid trials. We thus analyzed the data from the remaining 20 infants (9 females, mean age = 8.031 ± 0.252 months, Table S3, and Table S4).

#### Familiarization

Infants looked at the visual stimuli 73.8 ± 19.6% of the time, mainly the eyes area (98.5% of the time).

#### Test

The infants contributed on average with 10.2 ± 3.2 valid trials each during the test phase (range = 6 to 16). The time window for the analysis of the lateralized gaze was from 1140 to 4700 ms (at *P* < 0.01, Fig. 2e), and, again, infants looked to the expected regions of interest (Figure S1).

We found that the infants equally associated the words with the correct and the incorrect videos (mean TLT = 1464 ± 311 ms for the correct videos and 1596 ± 221 ms for the incorrect ones; *t*_(*19*)_ = − 1.363, *P* = 0.189), and the mean TLT-p, TLT-acc, LF-p, LF-acc were not different to chance (*t*_(*19*)_ = − 1.707, *P* = 0.104; *t*_(*19*)_ = − 2.092, *P* = 0.057; *t*_(*19*)_ = − 1.382, *P* = 0.183; and *t*_(*19*)_ = − 1.406, *P* = 0.176, respectively) (Fig. 2f). The FG was not different to chance (*t*_(19)_ = − 1.707, *P* = 0.096, Cohen’s d = − 0.381) and the accuracy did not significantly change during the test phase (see Figure S2).

Together, these results supported our predictions about the advantages of vowel over consonants guiding the learning of word-action associations from fluent audiovisual stimuli. To complete our evaluations in Experiment 4, we evaluated whether consonant harmony benefited the learning of word-object associations from fluent audiovisual streams.

### Experiment 4

This experiment emulated Experiment 2 but now added consonant harmony instead of vowel harmony to the object-words of the audiovisual streams (see Video S4). We evaluated 34 healthy infants, 11 were excluded, 5 because of fussiness and 6 because they contributed with less than 5 valid trials. We analyzed the data from the remaining 23 infants (11 females, mean age = 7.931 ± 0.199 months, Table S3, and Table S4).

#### Familiarization

The TLT-pct was 77.9 ± 13.6% showing that the infants explored the visual stimuli of the audiovisual stream and looked mainly at the eyes area (92.9% of the time).

#### Test

Infants contributed on average with 8.8 ± 3.8 valid trials each (range = 5 to 16). The time window for the analysis of the lateralized gaze was from 1120 to 4700 ms (at *P* < 0.01, Fig. 2g), starting at the last phoneme of the second presentation of the word. As in previous experiments, the infant gaze mainly fell into the expected regions of interest (Figure S1).

Similar to the Experiment 3, the infants equally looked at the correct and incorrect pictures when listening to the words (mean TLT = 1419 ± 196 ms for the correct picture and 1471 ± 225 ms for the incorrect one; *t*_(*22*)_ = − 1.203, *P* = 0.243). Moreover, the mean TLT-p, TLT-acc, LF-p and LF-acc were not different to chance (*t*_(*22*)_ = − 1.012, *P* = 0.323; *t*_(*22*)_ = − 0.136, *P* = 0.893; *t*_(*22*)_ = − 0.843, *P* = 0.408; and *t*_(*22*)_ = 0.451, *P* = 0.657, respectively) (Fig. 2h). No differences in FG were found (*t*_(22)_ = − 0.680, *P* = 0.503 Cohen’s d = − 0.142), and infants did not significant change their accuracy during the test phase (see Figure S2).

Consistent with our predictions, the consonant harmony did not facilitate the learning of word-object associations from fluent audiovisual stimuli.

To deeper explore the effect of vowels and consonant on the early steps of word learning, we proceeded to compare the results across the experiments.

### Cross-experiments analysis

Because all the experiments pursued similar goals, applied almost identical protocols, measured the same variables, and evaluated infants with a similar bio-demographic background (Table S3, and Table S4), we analyzed the data across the experiments. This analytic approach allowed us to draw more mature conclusions about the role of phonological cues on the learning of new word-images associations from fluent contexts during early infancy.

#### Phonological cues

We submitted the test's visual variables to a two-ways ANOVA with type of phonemic cue (Vowel harmony & Consonant harmony) and type of evaluated word (Action-word & Object-word) as between-subject factors. We found a main effect of the type of phonemic cue, because the mean TLT-p, TLT-acc, LF-p and LF-acc were significantly greater when the words conveyed vowel harmony than consonant harmony (*F*_(1,83)_ = 14.694; *P* < 0.001; ŋ2p = 0.155; *F*_(1,83)_ = 16.170; *P* < 0.001; ŋ2p = 0.168; *F*_(1,83)_ = 10.167; *P* = 0.002; ŋ2p = 0.113; and *F*_(1,83)_ = 16.325; *P* < 0.001; ŋ2p = 0.169, respectively). There were not significant type of phonemic cue X type of evaluated word interactions.

A possible explanation for such phoneme asymmetry could relate to age. Indeed, previous studies reported that during the first six months of age, infants are more sensitive to vowels than consonants to encode and recall new words learned in non-fluent contexts^11–13^. The sensitivity to consonants for word learning would emerge in the second semester of age, in languages such as French^13,24^ and English^25,26^. To some extent, our data support this proposal by showing that, only in the experiments providing consonant harmony (Experiment 3 and Experiment 4 together), we found that the greater the age, the greater the TLT-p (Pearson’s r = 0.336, *P* = 0.027, n = 43) and the TLT-acc (Pearson’s r = 0.386, *P* = 0.011, n = 43). The same correlations against age were not significant in the experiments that provided vowel harmony as a phonological cue (*P* > 0.420) (Figure S3). Moreover, the results of the ANCOVA adding the infant’s age as a co-variable, were similar to the one observed in the ANOVA (Table S6), confirming that the differences in infant’s age did not explain the differences we found for visual variables between vowel and consonant harmony.

#### Infant’s engagement with the task

Importantly, the groups did not differ in the total looking time exploring the video during familiarization (*P* = 0.099) or in the number of valid trials (*P* = 0.468), confirming that the infants equally engaged with the task across the experiments.

#### No effect of bio-demographic data

Additionally, the participants of all experiments were highly similar in bio-demographic measures (see methods). A series of multiple regression analyses showed that the bio-demographic factors did not explain the variance in total looking time during familiarization (Table S4 and Figure S4) across experiments. Moreover, the regression analyses of the test's data showed that the phonemic cue was the main factor explaining the variance we found in word-image learning, without any significant effect of the number of the valid trials or any bio-demographic factors we measured (Table S7 to Table S11).

## Discussion

Our results showed that 8-month-old infants were able to extract both, object- and action-words from continuous audiovisual streams. However, infants succeeded in the task only when words conveyed vowel harmony as a phonological cue. To our knowledge, this infant's ability remained hidden until now.

Our results contrast with those reported in a previous study, which directly explored the subject in 8-month-old infants^4^. In the mentioned study, the trisyllabic words co-occurred with geometric figures. The results showed that although infants discovered the words they failed to learn their associations with the images. The authors suggested that, at this age, infants had a bias to process speech over visual stimuli.

Our data showed that vowel harmony facilitated the word-visual referent associations. In agreement with previous data showing that vowel harmony facilitates fluent speech segmentation in 7-months-old^14^, we may propose that infants exploited vowel harmony to segment the speech track of the audiovisual stream and to discover the words. After finding the words, the infants could have implemented the mechanisms to link the words with the corresponding images and probably saved in memory as multi-sensorial representations for further recall. Words discovery may have allowed word-image associations learning. Indeed, in infants before the first year of age, labels benefit a number of nonlinguistic representations such as object representation^27^ and object category formation^28^. Nevertheless, we cannot discard the possibility that the learning of word-visual referent associations we observed here could have been facilitated by the information provided by the visual cues or by the multimodal processing. Indeed, the images synchronically switched at the word’s edges, providing a powerful segmentation clue. Also, the multimodal processing of speech and images may have prompted infants into a cognitive state where vowel harmony could be better exploited to learn word-image associations. Further studies are necessary to explore those possibilities more deeply.

In contrast, consonant harmony did not benefit the learning of word-image association, perhaps because consonant perception requires greater auditory skills. To some extent, our results agree with previous studies about word learning from non-fluent contexts. These studies reported that before 6 months of age, infants mainly exploit vowels to memorize new words^11–13^. Only near their first birthday infants exploit more consonants than vowels to learn new words^12^.

The nature of the developmental asymmetry to exploit vowels and consonants for word learning during infancy is not fully understood. However, it may emerge from their acoustic properties. First, vowels are acoustically more salient than consonants because they carry the energy, pitch variability, duration of the speech^24^, and ultimately prosody^29^. Thus vowels may act as better perceptual and attentional attractors. Previous studies showed that attention benefits word-object learning during early infancy^30^. In our study, allocating the attention to the salient parts of the speech component of the audiovisual stream may benefit the segmentation of the stream, the discovery of the words, and consequently, the encoding of the word-image associations.

Second, previous studies indicate that we perceive vowels as slow changes in the vocalizations consonants provide a wide range of rapid changes that emerged from the articulators and constriction in the vocal tract. Curiously, similar advantage of vowels over consonants for speech intelligibility is observed in older adults with hearing difficulties^31^. Consonant processing may thus require more advanced neurocognitive maturation or greater experience with speech to guide speech segmentation task or word-image association encoding. Interestingly, although at the chance level and within a tight age range, we found that in the two experiments that provided consonant harmony, the accuracy in word-image associations learning positively correlated with age.

A crucial result of our study is the fact that infants learned both object- and action-words. To some extent, we may state that 8-month-olds could discover words with different properties, some of them generalized across two women and others associated to a unique face. This distinction of the type of word may unveil the learning of a basic notion of lexical categories. However, this ability to recognize types of words was associated with our experimental design because we intentionally trained infants to generalize the action-words across two women, and to associate a single face to a particular object-word. Further studies are thus necessary to evaluate whether infants could also associate many images of the same woman with a single object-word, relying on generalization processing about individuals. Previous studies have suggested that the learning of word-visual referents associations before the first year unveil a rudimentary version of nouns and verbs^32^. Our results ultimately may provide new data about the very early capacities of human infants encoding nouns and verbs as different lexical categories.

Longitudinal studies are necessary to explore whether the early abilities we report here relate to the success in later stages of language acquisition. Indeed, in toddlers, the learning of word-visual referent associations positively correlates with productive vocabulary size^33,34^.

The social cues that we added to our stimuli may have contributed to the learning by increasing the engagement in the task. We used smiling faces with a direct gaze and familiar gestures instead of geometric figures. Previous studies have shown that smiling faces with direct gaze are powerful cues to inform young infants that the stimulation is directed to them^35^, and in 9-months-old enhance the learning of basic events from uncertain and noisy environments^36^. Moreover, 8-months-old learn better the label associated with a novel object when the object makes familiar movements than when they move with unusual ones^37,38^, and 2-year-olds map familiar objects to novel labels more easily than unfamiliar objects^39^. While we used faces and yes-like and no-like head movements, we provided infants with a highly familiar context against which they could match the words. Together, socially relevant familiar visual events may have facilitated the statistical learning^4^.

Nevertheless, the contribution of social cues to the task could not explain the difference in our results, because they were provided identically in each one of the four experiments, including those when the infants failed to make the word-image associations.

Our study has several limitations that require further studies to be clarified. First, we compared experimental conditions across groups, and in each experiment, we measured the learning of only 2 from the 4 words, i.e. those conveying the phoneme harmony. This protocol was used to reduce the infants’ cognitive demands because the learning of new word-visual referent associations learning at the laboratory has been described as hard even in non-fluent contexts^40^. Previous studies in young infants reported that more complex tasks such as word-object categorization are accompanied by lower accuracy and gaze’s long latency starting 1.5 s after the onset of the object presentation^40^. The long latency to switch the gaze toward the selected image we observed in all our experiments supports this idea.

Important to notice that given that the task consisted of learning novel words, we were unable to measure eventual a-priori preferences for each of them. However, we controlled this potential bias to prefer particular words, faces, head gestures, or word-image pairs by counterbalancing those items across infants (see Table S1). Moreover, we did not find preferences for any item in our analysis.

Furthermore, the participants across the groups were very similar in their visual behavior. They showed a similar interest to explore the images during the familiarization and test phases, contributed with a similar number of valid test trials, and showed similar bio-demographic data.

In any case, further intra-subject designs would be necessary to better understand the asymmetric contribution of vowel and consonant harmony on word-visual referent association learning. In sighted and hearing infants, word-learning is primarily an intermodal process involving the mapping between a speech signal and a visual referent^1,41^, and considerable effort has been done to explore the mechanisms underpinning such process during early development. Although word learning exceeds the ability to make audiovisual associations^1,42^, the discovery of word-image associations from fluent scenes during early infancy may be crucial in shaping later stages of word learning and lexical category learning during language acquisition. We showed that vowels seem to be crucial in facilitating the encoding of word-visual referent associations from fluent audiovisual stimuli, possibly operating through prosody modulations. Moreover, we showed that vocalic information equally benefits the learning of words associated with objects and actions, uncovering the infants' capacities to succeed in encoding not only the words but also primitive versions of lexical categories. Our results may thus serve as inputs to fit the current models of word-learning during early infancy.

## Methods

All methods were carried out in accordance with relevant guidelines and regulations.

### Infant recruitment and ethics

The sample size was estimated taking into account previous behavioral studies on eye tracking^43^ and a predicted moderate effect size of 0.6 for the confidence level of 95% (G-power estimate for the one-sample t-test). We recruited infants in public primary health care centers, where infants regularly attend for preventive control.

The research reported in this manuscript has been conducted in accordance with the principles expressed in the Declaration of Helsinki and approved by the local ethical committee (The Social Science Ethics Committee of the Pontifical Catholic University of Chile). All parents signed an informed written consent for their infants to participate in this study.

### Participants and participants’ demographic data

All infants belonged to a middle-low socioeconomic class, monolingual Spanish-speaking environment, and presented a history of typical physical, psychomotor, and sensorial development at the test time. None had a history of specific language impairment or family dyslexia. We purposefully excluded from the study infants with any disease that per se causes or predisposes to developmental alterations, including neonatal asphyxia, epilepsy, chromosomal disorders, and inborn errors of metabolism. Additionally, all mothers scored a low-stress level in the Parental Stress Index test, screening version, indicating similar quality in mother-infant interaction.

During the experimental session, the parents provided information about: a) infant’s birth data; b) mother’s age; c) maternal education level, where level 1 corresponded to incomplete elementary and middle school (less than 8 years), level 2 to full elementary and middle school (8 years), level 3 to incomplete high school (less than 4 years), level 4 to full high school (4 years), 5 = incomplete college, 6 = Bachelor degree, 7 = post-graduate training; d) months of exclusive breastfeeding; e) the age in months at which the infant presented the first social smile, first babbling and seated alone, these three abilities are used as screening of psychomotor development at the local level. None of the data we reported here allows the identification of the participants.

### Stimuli

#### Auditory stimuli

Each speech stream used in the familiarization phase was built by concatenating 4 nonsense trisyllabic words (i.e., CVCVCV), containing 24 presentations of each word. We generated a monotonous speech stream by using MBROLA, a text-to-speech synthesizer (The MBROLA project, http://tcts.fpms.ac.be/synthesis/mbrola.html) using fr4 diphone database, 200 Hz pitch (i.e., flat prosody), 170 ms length for any consonant, and 286 ms for any vowel. TP between adjacent phonemes within words were equal to 1, and between words were equal to 0.5, along any speech stream. The list of words for the streams in each experiment is in Table S1. By using EsPal database^44^, we confirmed that any consecutive trisyllabic chunk of the streams was not a real word in Spanish and did not have phonological neighbors. We controlled as much as we could the frequency of the CV bigrams at the first, second and third syllables in Spanish of the target and non-target words in all experiments. The mean frequency of CV bigram was lower in the experiments with vowel harmony than in the experiments with consonant harmony, however, the pattern of differences in frequency at each syllabic position was similar between them (Figure S5).

#### Visual stimuli

We first recorded short videos of the two women separately. We asked them to stay quiet and move their heads while smiling and maintaining a direct gaze all the time. We then extracted pictures and videos of head gestures and equalized them in duration, gest speed, and luminance. All images were in color. Each one of the women appearing in the images of this study signed an informed consent authorizing the use of their images in an online open-access publication.

#### Audiovisual stream

We built the audiovisual stream by synchronizing the speech to the visual track. In the stream, two words synchronically appeared with pictures, one per woman, and the other two words co-occurred with the videos, one per head gesture, each one made by both women. Moreover, the object-words preceded the action-word, in a way that both, object- and action-words were made by the same woman. We thus facilitated the fluency in delivering the audiovisual information across the stream.

To avoid eventual biases for particular faces, gestures, words, and word-image pairs we counterbalanced all stimuli across experiments and infants. We created 3 audiovisual streams per experiment, each stream contained different word-images pairs made by different women. Infants were randomly evaluated with one of those versions (Table S1).

### Procedure

We conducted the study in a soundproof and dimly lit room without any distraction. The infants were tested on their caregiver’s lap at 60 cm in front of the eye-tracker monitor. Parents were instructed not to intervene in the infant’s behavior and wore dark glasses to prevent them from seeing the visual stimuli.

We displayed the visual stimuli on a 17-in. Eye-tracker monitor with 1024 × 768 pixels and 16-bit color depth (Tobii 1705). The tracker remotely recorded the infant’s binocular eye fixations with a sampling rate of 50 Hz (i.e., 20 ms) and parsed data into fixations and saccades. The speech was played through a hidden loudspeaker at 60 dB.

We first calibrated the infant’s binocular gaze using fixations longer than 100 ms on five centered points and the four corners of the monitor.

The experiment had two phases, familiarization and test. The familiarization phase presented the continuous audiovisual stream in the center of the screen, with a size of 550 × 580 pixels of resolution, subtending an 11.6º × 19.2º area. The background was always grey. Once the familiarization ended, the test phase started, pseudorandomly presenting 8 trials, which were repeated twice, obtaining 16 trials in total. Each trial started with an audiovisual attractor to attract the visual infant's attention to the center of the screen, subtending a 2.9º × 2.9º area. We then presented an image with two lateralized light grey squares, one on each side of the screen, where the stimuli will be displayed with a size of 380 × 350 pixels subtended a 11º × 12.7º area each. After 1 s of silence, we played the same word twice, separated by 1 s of silence, but now each word was synchronized with the presentation of an image containing two visual stimuli (picture or videos), replacing each empty square, with only one matching the presented auditory word. The side of the correct image was counterbalanced across trials and infants. The experiment was automatized by using PsyScope X (http://psy.cns.sissa.it).

### Eye-tracking recordings

Tobii automatically parses data into fixations and saccades. Fixation detection was computed by applying a standard dispersal-based algorithm in Clearview 2.7 (Tobii Eye Tracker) with a dispersal threshold of 30 pixels (corresponding to 0.9°) and a minimum temporal duration of 100 ms.

### Data-driven approach for time window analysis

Time windows for the analysis of the test phase were calculated using a data-driven approach. First, in each valid trial of each infant, we computed the position of the gaze over the horizontal axis (in pixel) at each time point (every 20 ms), generating thus a time course of how the gaze moved across the horizontal axis. We then transformed this time course into a binary value, by assigning 1 to every time point when the position of the gaze fell over the lateralized images and 0 otherwise. We then averaged the binary time course across the valid trials in each infant. Finally, we submitted the averages of all infants to a one-sample t-test (right tail, alpha = 0.01) against 0.98, where 0.98 indicated that we required that the gaze at each time point was significantly greater than 0.98 to be considered significantly lateralized, at *P* < 0.01.

## Supplementary Information

Supplementary Information 1.

Supplementary Video 1.

Supplementary Video 2.

Supplementary Video 3.

Supplementary Video 4.

## References

[1] Waxman SR, Lidz J, Kuhn D, Siegler R (2006). Early word learning. Handbook of Child Psychology.

[2] Saffran JR, Aslin RN, Newport EL (1996). Statistical learning by 8-month-old infants. Science.

[3] Kirkham NZ, Slemmer JA, Johnson SP (2002). Visual statistical learning in infancy: Evidence for a domain general learning mechanism. Cognition.

[4] Thiessen ED (2010). Effects of visual information on adults’ and infants’ auditory statistical learning. Cogn. Sci..

[5] Tincoff R, Jusczyk P (1999). Some beginnings of word comprehension in 6-month-olds. Psychol. Sci..

[6] Tincoff R, Jusczyk P (2012). Six-month-olds comprehend words that refer to parts of the body. Infancy.

[7] Tardif T, Fletcher P, Liang W, Zhang Z, Kaciroti N, Marchman VA (2008). Baby's first 10 words. Dev. Psychol..

[8] Friedrich M, Friederici AD (2017). The origins of word learning: brain responses of 3-month-olds indicate their rapid association of objects and words. Dev. Sci..

[9] Junge C, Cutler A, Hagoort P (2012). Electrophysiological evidence of early word learning. Neuropsychologia.

[10] Gogate LJ, Hollich GJ (2010). Invariance detection within an interactive system: A perceptual gateway to language development. Psychol. Rev..

[11] Benavides-Varela S, Hochmann JR, Macagno F, Nespor M, Mehler J (2012). Newborn’s brain activity signals the origin of word memories. Proc. Natl. Acad. Sci. U.S.A..

[12] Hochmann JR, Benavides-Varela S, Nespor M, Mehler J (2011). Consonants and vowels: different roles in early language acquisition. Dev. Sci..

[13] Nishibayashi LL, Nazzi T (2016). Vowels, then consonants: early bias switch in recognizing segmented word forms. Cognition.

[14] Mintz TH, Walker RL, Welday A, Kidd C (2018). Infants' sensitivity to vowel harmony and its role in segmenting speech. Cognition.

[15] Wang P, Gauthier I, Cottrell G (2016). Are face and object recognition independent? a neurocomputational modeling exploration. J. Cogn. Neurosci..

[16] Barry C, Johnston RA, Scanlan LC (1998). Are faces "special" objects? Associative and semantic priming of face and object recognition and naming. Q. J. Exp. Psychol. A Hum. Exp. Psychol..

[17] Morton J, Johnson MH (1991). CONSPEC and CONLERN: a two-process theory of infant face recognition. Psychol. Rev..

[18] Valenza E, Simion F, Macchi Cassia V, Umiltà C (1996). Face preference at birth. J. Exp. Psychol. Hum. Percept. Perform..

[19] Bonatti L, Frot E, Zangl R, Mehler J (2002). The human first hypothesis: Identification of conspecifics and individuation of objects in the young infant. Cogn. Psychol..

[20] Gogate LJ, Bahrick LE, Watson JD (2000). A study of multimodal motherese: the role of temporal synchrony between verbal labels and gestures. Child Dev..

[21] Wass SV, Prior J, Herwegen JV (2016). The use of eye-tracking with infants and children. Practical Research with Children.

[22] Aslin RN (2007). What's in a look?. Dev. Sci..

[23] Fagan JF, McGrath SK (1981). Infant recognition memory and later intelligence. Intelligence.

[24] Bouchon C, Floccia C, Fux T, Adda-Decker M, Nazzi T (2015). Call me Alix, not Elix: vowels are more important than consonants in own-name recognition at 5 months. Dev. Sci..

[25] Floccia C, Nazzi T, Luche CD, Poltrock S, Goslin J (2014). English-learning one- to two-year-olds do not show a consonant bias in word learning. J. Child Lang..

[26] Mani N, Plunkett K (2007). Phonological specificity of vowels and consonants in early lexical representations. J. Mem. Lang..

[27] Twomey KE, Westermann G (2018). Learned labels shape pre-speech infants’ object representations. Infancy.

[28] Fulkerson AL, Waxman SR (2007). Words (but not tones) facilitate object categorization: Evidence from 6- and 12-month-olds. Cognition.

[29] Nespor M, Peña M, Mehler J (2003). On the different roles of vowels and consonants in speech processing and language acquisition. Lingue e Linguaggio.

[30] Gogate LJ, Maganti M (2016). The dynamics of infant attention: Implications for crossmodal perception and word-mapping research. Child Dev..

[31] Fogerty D, Kewley-Port D, Humes LE (2012). The relative importance of consonant and vowel segments to the recognition of words and sentences: effects of age and hearing loss. J. Acoust. Soc. Am..

[32] Gogate L, Hollich G (2016). Early verb-action and noun-object mapping across sensory modalities: a neuro-developmental view. Dev. Neuropsychol..

[33] Katerelos M, Poulin-Dubois D (2011). A longitudinal study of word learning: interrelations between word-event association, fast mapping, and vocabulary. Dans Enfance.

[34] Gershkoff-Stowe L, Hahn ER (2007). Fast mapping skills in the developing lexicon. J. Speech Lang. Hear. Res..

[35] Csibra G, Gergely G (2009). Natural pedagogy. Trends Cogn. Sci..

[36] Wu R, Kirkham NZ (2010). No two cues are alike: depth of learning during infancy is dependent on what orients attention. J. Exp. Child Psychol..

[37] Matatyaho DJ, Gogate L, Mason Z, Cadavid S, Abdel-Mottaleb M (2014). Type of object motion facilitates word mapping by preverbal infants. J. Exp. Child Psychol..

[38] Fennell C (2011). Object familiarity enhances infants’ use of phonetic detail in novel words. Infancy.

[39] Hall GD (1991). Acquiring proper nouns for familiar and unfamiliar objects: two-year-olds’ word-learning biases. Child Dev..

[40] Taxitari L, Twomey KE, Westermann G, Mani N (2019). The limits of infants' early word learning. Lang. Learn. Dev..

[41] Hirsh-Pasek K, Golinkoff RM (1996). The Origin of Grammar.

[42] Samuelson L, McMurray B (2017). What does it take to learn a word? Wiley interdisciplinary reviews. Cogn. Sci..

[43] Kovács AM, Mehler J (2009). Cognitive gains in 7-month-old bilingual infants. Proc. Natl. Acad. Sci. U.S.A..

[44] Duchon A, Perea M, Sebastián-Gallés N, Martí A, Carreiras M (2013). EsPal: One-stop shopping for Spanish word properties. Behav. Res..

