# Supplementary material for: Infants exploit vowels to label objects and actions from continuous audiovisual stimuli
Source: Sci Rep. 2021 May 26;11:10982. doi: 10.1038/s41598-021-90326-z (PMC8154951; doi:10.1038/s41598-021-90326-z)
Supplement: Supplementary file 1 — Supplementary Information 1. [file 41598_2021_90326_MOESM1_ESM.pdf]

Supplementary Information

Infants exploit vowels to label objects and actions from continuous audiovisual stimuli.

Cristina Jara[1,2]*, Cristóbal Moënne-Loccoz[2], Marcela Peña[1]*

Supplementary tables

Table S1. Words and images. To prevent for eventual item-related bias, in the familiarization and test we counterbalanced the word-object and word-action pairs across infants. We detail the list of the words and images used to build the different versions of the audiovisual continuous streams presented to different infants in each experiment. The words evaluated in the test phase per experiment are presented in bold. Words are expressed as Spanish diphones.

		**Visual stimuli**			
		**Woman 1** (object-word)	**Woman 2** (object-word)	**Up-and-down** (action-word)	**Left-to-right** (action-word)
**Experiment 1**	List 1 (n = 6)	tofanu	puliso	**mibiki**	**dagava**
	List 2 (n = 8)	puliso	tofanu	**mibiki**	**dagava**
	List 3 (n = 9)	tofanu	puliso	**dagava**	**mibiki**
**Experiment 2**	List 1 (n = 6)	**mibiki**	**dagava**	tofanu	puliso
	List 2 (n = 9)	**dagava**	**mibiki**	tofanu	puliso
	List 3 (n = 6)	**dagava**	**mibiki**	puliso	tofanu
**Experiment 3**	List 1 (n = 7)	meboki	digave	**nonina**	**lalelo**
	List 2 (n = 7)	digave	meboki	**nonina**	**lalelo**
	List 3 (n = 6)	digave	meboki	**lalelo**	**nonina**
**Experiment 4**	List 1 (n = 6)	**nonina**	**lalelo**	meboki	digave
	List 2 (n = 10)	**lalelo**	**nonina**	meboki	digave
	List 3 (n = 7)	**lalelo**	**nonina**	digave	meboki

Table S2. All data had a normal distribution, proved by submitting each data to the Shapiro-Wilk test. We report the W-value and P-value for each dependent variable in each experiment.

	**Experiment 1**<br>**n = 23**	**Experiment 2**<br>**n = 21**	**Experiment 3**<br>**n = 20**	**Experiment 4**<br>**n = 23**
**TLT-acc**	$W_{(22)} = 0.951$<br>$P = 0.310$	$W_{(20)} = 0.936$<br>$P = 0.180$	$W_{(19)} = 0.911$<br>$P = 0.068$	$W_{(22)} = 0.943$<br>$P = 0.204$
**TLT-p**	$W_{(22)} = 0.952$<br>$P = 0.318$	$W_{(20)} = 0.959$<br>$P = 0.504$	$W_{(19)} = 0.970$<br>$P = 0.750$	$W_{(22)} = 0.954$<br>$P = 0.352$
**LF-acc**	$W_{(22)} = 0.958$<br>$P = 0.433$	$W_{(20)} = 0.936$<br>$P = 0.180$	$W_{(19)} = 0.94$<br>$P = 0.235$	$W_{(22)} = 0.961$<br>$P = 0.483$
**LF-p**	$W_{(22)} = 0.977$<br>$P = 0.855$	$W_{(20)} = 0.974$<br>$P = 0.818$	$W_{(19)} = 0.94$<br>$P = 0.235$	$W_{(22)} = 0.973$<br>$P = 0.753$
**FG-acc**	$W_{(22)} = 0.971$<br>$P = 0.722$	$W_{(20)} = 0.956$<br>$P = 0.434$	$W_{(19)} = 0.968$<br>$P = 0.723$	$W_{(22)} = 0.967$<br>$P = 0.610$

Table S3. Demographic data**.** We report **the** mean and standard deviations (SD) for demographic data per experiment, and the ANOVA statistics corresponding to the comparison between experiments. We did not find differences in any comparison ($P > 0.169$).

	**Experiment 1 n = 23**		**Experiment 2 n = 21**		**Experiment 3 n = 20**		**Experiment 4 n = 23**		**ANOVA**
	**Mean**	**SD**	**Mean**	**SD**	**Mean**	**SD**	**Mean**	**SD**	**Stats**
Infants' age (months)	7.962	0.445	7.964	0.238	8.031	0.253	7.932	0.199	$F_{(3, 83)} = 0.405$ $P = 0.750$
Gestational Age (weeks)	39.087	0.900	38.667	1.197	38.650	0.988	39.196	0.914	$F_{(3, 83)} = 1.722$ $P = 0.169$
Mothers' age (years)	28.913	7.135	29.810	8.704	29.000	6.061	29.304	6.540	$F_{(3, 83)} = 0.068$ $P = 0.977$
Weight at birth (gr)	3416	457	3303	486	3353	450	3438	505	$F_{(3, 83)} = 0.363$ $P = 0.780$
Height at birth(cm)	50.130	1.448	49.143	2.372	49.615	2.028	49.196	2.199	$F_{(3, 83)} = 1.141$ $P = 0.337$
APGAR 5th min	9.048	0.590	8.950	0.224	8.900	0.308	9.000	0.000	$F_{(2, 58)} = 0.694$ $P = 0.504$
First babbling (months)	4.045	1.253	3.850	1.725	4.225	1.543	4.565	1.199	$F_{(3, 83)} = 0.978$ $P = 0.407$
First social smile (months)	2.826	0.112	3.143	1.062	3.450	1.146	3.565	1.532	$F_{(3, 83)} = 1.468$ $P = 0.229$
Seated alone (months)	5.087	0.625	5.476	1.030	5.250	0.967	5.174	1.230	$F_{(3, 83)} = 0.462$ $P = 0.709$
Breastfeeding (months)	5.174	2.443	5.000	2.550	4.250	3.193	4.409	3.003	$F_{(3, 83)} = 0.548$ $P = 0.651$

Table S4. Infants' gender and mothers' educational level distribution per experiment. # = number and % = percentage from the total. The infants from different experiments did not differ in any measures (Chi-square with $P > 0.707$ for any comparison).

		Experiment 1 n = 23		Experiment 2 n = 21		Experiment 3 n = 20		Experiment 4 n = 23		Chi-square
		#	%	#	%	#	%	#	%	Stats
**Infant Gender**	Male	12	52.174	10	47.619	11	55	11	47.826	$X^2_{(3,87)} = 0.323 P = 0.956$
	Female	11	47.826	11	52.381	9	45	12	52.174	
**Mother' educational level**	Middle school, incomplete	0	0.000	1	4.762	0	0.000	0	1.149	$X^2_{(18, 87)} = 14.342$ $P = 0.707$
	Middle school, complete	2	8.696	0	0.000	0	0.000	3	5.747	
	High school, incomplete	2	8.696	4	19.048	2	10.000	3	12.644	
	High school complete	8	34.783	7	33.333	8	40.000	8	35.632	
	College incomplete,	4	17.391	1	4.762	3	15.000	2	11.494	
	Bachelor degree	7	30.435	7	33.333	7	35.000	7	32.184	
	Not informed	0	0.000	1	4.762	0	0.000	0	1.149	

Table S5. We did not observe any item bias on the correct responses due to preferences for particular words, head gesture or woman in any experiment. We describe the statistics for Experiment 1 and Experiment 2, which exhibited performances significantly above chance.

			Correct trials	Incorrect trials	Stats
**Experiment 1**	action-words	dagava	56	42	$X^2_{(1,200)} = 0.01$, $P = 0.920$
		mibiki	59	43	
	head gesture	woman 1	59	43	$X^2_{(1,200)} = 0.01$, $P = 0.920$
		woman 2	56	42	
**Experiment 2**	object-word	dagava	56	44	$X^2_{(1,193)} = 0.807$, $P = 0.368$
		mibiki	58	35	
	Picture	woman 1	62	40	$X^2_{(1,193)} = 0.264$, $P = 0.607$
		woman 2	52	39	

Table S6. The results of the ANCOVA with infant' age as co-variable, were very similar to the results of the ANOVA for the cross-experiments comparisons. We indicate the mean, standard deviation, and n for vowel and consonant harmony phonemic cues.

**Variable**	**Phonemic cue**	**ANCOVA**	**ANOVA**
TLT-acc	Vowel harmony	*F*(183) = 13.906 *P* < 0.001 n2p = 0.143	*F*(183) = 14.694 *P* < 0.001 n2p = 0.155
	0.591 ± 0.1642 (n = 44)		
	Consonant harmony		
	0.466 ± 0.151 (n = 43)		
TLT-p	Vowel harmony	*F*(183) = 15.88 *P* < 0.001 n2p = 0.16	*F*(183) = 16.17 *P* < 0.001 n2p = 0.168
	0.544 ± 0.083 (n = 44)		
	Consonant harmony		
	0.480 ± 0.066 (n = 43)		
LF-acc	Vowel harmony	*F*(183) = 8.35 *P* = 0.005 n2p = 0.09	*F*(183) = 10.167 *P* = 0.002 n2p = 0.113
	0.584 ± 0.164 (n = 44)		
	Consonant harmony		
	0.489 ± 0.148 (n = 43)		
LF-p	Vowel harmony	F(183) = 17.01 *P* < 0.001 n2p = 0.17	*F*(183) = 16.325 *P* < 0.001 n2p = 0.169
	0.544 ± 0.07 (n = 44)		
	Consonant harmony		
	0.484 ± 0.065 (n = 43)		

Table S7: Multiple regression analysis for TLT-pct during familiarization. Effect of bio-demographic data and Experiment. The model was not significant, although Maternal Education explained part of the variance in TLT-pct (see Figure S3).

MODEL INFO:

Observations: 87

Dependent Variable: TLT-pct

Type: OLS linear regression

MODEL FIT:

$F(11,75) = 1.478, p = 0.158$

$R^2 = 0.178$

Adj. $R^2 = 0.058$

Standard errors: OLS

	Est.	S.E.	t val.	P
(Intercept)	91.313	56.282	1.622	0.109
Experiment2	2.740	5.840	0.469	0.640
Experiment3	9.742	5.972	1.631	0.107
Experiment4	10.010	5.785	1.712	0.091
Mother's age	-0.359	0.305	-1.178	0.243
Maternal education	-4.778	1.785	-2.676	0.009**
Breastmilk	-0.549	0.763	-0.720	0.474
Infant's age	0.433	7.140	0.061	0.952
GenderMale	-4.610	4.401	-1.047	0.298
First social smile	0.232	1.700	0.137	0.892
First babling	-0.736	1.642	-0.449	0.655
Seated alone	1.832	2.081	0.881	0.381

Est. = estimator; S. E. = standard error; t val. = t value; p = probability. * = p <0.05; ** = p < 0.01; *** = p < 0.001

Table S8: Multiple regression analysis for the TLT-acc in the test phase. Effect of bio-demographic factors and Experiment. The model was significant, because the Experiment 3 and 4 had lower TLT-acc than the Experiment 1.

MODEL INFO:

Observations: 87

Dependent Variable: TLT-acc

Type: OLS linear regression

MODEL FIT:

*$F(11,75) = 2.611$, $p = 0.007$***

$R^2 = 0.277$

Adj. $R^2 = 0.171$

Standard errors: OLS

	Est.	S.E.	t val.	p
(Intercept)	0.129	0.456	0.282	0.778
Experiment2	0.002	0.047	0.052	0.959
Experiment3	-0.170	0.048	-3.519	0.001**
Experiment4	-0.126	0.047	-2.691	0.009**
Mother's age	0.000	0.002	0.148	0.883
Maternal education	-0.018	0.014	-1.245	0.217
Breastmilk	0.006	0.006	0.890	0.376
Infant's age	0.056	0.058	0.976	0.332
GenderMale	-0.031	0.036	-0.874	0.385
First social smile	0.024	0.014	1.762	0.082
First babling	0.025	0.013	1.853	0.068
Seated alone	-0.019	0.017	-1.112	0.269

Est. = estimator; S. E. = standard error; t val. = t value; p = probability. * = p <0.05; ** = p < 0.01; *** = p < 0.001

Table S9: Multiple regression analysis for the TLT-p in the test phase. Effect of bio-demographic factors and Experiment. The model was significant, because the Experiment 3 and 4 had lower TLT-p than Experiment 1.

MODEL INFO:

Observations: 87

Dependent Variable: TLT-p

Type: OLS linear regression

MODEL FIT:

*$F(11,75) = 2.872$, $p = 0.003$***

$R^2 = 0.296$

Adj. $R^2 = 0.193$

Standard errors: OLS

	Est.	S.E.	t val.	p
(Intercept)	0.291	0.217	1.340	0.184
Experiment2	-0.002	0.023	-0.103	0.918
Experiment3	-0.079	0.023	-3.422	0.001**
Experiment4	-0.071	0.022	-3.165	0.002**
Mother's age	0.001	0.001	0.970	0.335
Maternal education	-0.011	0.007	-1.571	0.120
Breastmilk	0.004	0.003	1.264	0.210
Infant's age	0.027	0.028	0.965	0.338
GenderMale	-0.021	0.017	-1.243	0.218
First social smile	0.016	0.007	1.364	0.188
First babling	0.006	0.006	0.928	0.357
Seated alone	-0.004	0.008	-0.458	0.648

Est. = estimator; S. E. = standard error; t val. = t value; p = probability. * = p <0.05; ** = p < 0.01; *** = p < 0.001

Table S10: Multiple regression analysis for the LF-acc in the test phase. Effect of bio-demographic factors and Experiment. Although the model did not reach significance, the longest fixation was significantly smaller in Experiment 3 and 4 than in Experiment 1.

MODEL INFO:

Observations: 87

Dependent Variable: LF-acc

Type: OLS linear regression

MODEL FIT:

$F(11,75) = 1.350$, $p = 0.215$

$R^2 = 0.165$

Adj. $R^2 = 0.043$

Standard errors: OLS

	Est.	S.E.	t val.	p
(Intercept)	0.777	0.472	1.648	0.103
Experiment2	0.008	0.049	0.166	0.869
Experiment3	-0.122	0.050	-2.434	0.017*
Experiment4	-0.083	0.048	-2.073	0.042*
Mother's age	0.003	0.003	1.109	0.271
Maternal education	-0.010	0.015	-0.700	0.486
Breastmilk	0.001	0.006	0.216	0.829
Infant's age	-0.039	0.060	-0.649	0.519
GenderMale	-0.035	0.037	-0.963	0.339
First social smile	0.014	0.014	0.973	0.334
First babling	0.010	0.014	0.721	0.473
Seated alone	0.001	0.017	0.079	0.938

Est. = estimator; S. E. = standard error; t val. = t value; p = probability. * = p <0.05; ** = p < 0.01; *** = p < 0.001

Table S11: Multiple regression analysis for the LF-p in the test phase. Effect of bio-demographic factors and Experiment. The model was significant because the longest fixation proportion was significantly smaller in Experiment 3 and 4 than in Experiment 1.

MODEL INFO:

Observations: 87

Dependent Variable: LF-p

Type: OLS linear regression

MODEL FIT:

*$F(11,75) = 2.425$, $p = 0.012$**

$R^2 = 0.262$

Adj. $R^2 = 0.154$

Standard errors: OLS

	Est.	S.E.	t val.	p
(Intercept)	0.364	0.201	1.812	0.074
Experiment2	0.013	0.021	0.621	0.536
Experiment3	-0.060	0.021	-2.800	0.007**
Experiment4	-0.055	0.021	-2.650	0.010*
Mother's age	0.002	0.001	1.770	0.081
Maternal education	-0.007	0.006	-1.093	0.278
Breastmilk	0.003	0.003	1.104	0.273
Infant's age	0.012	0.025	0.489	0.626
GenderMale	-0.007	0.016	-0.416	0.679
First social smile	0.009	0.006	1.471	0.146
First babling	0.002	0.006	0.358	0.722
Seated alone	0.000	0.007	0.065	0.948

Est. = estimator; S. E. = standard error; t val. = t value; p = probability. * = p <0.05; ** = p < 0.01; *** = p < 0.001

Supplementary figures

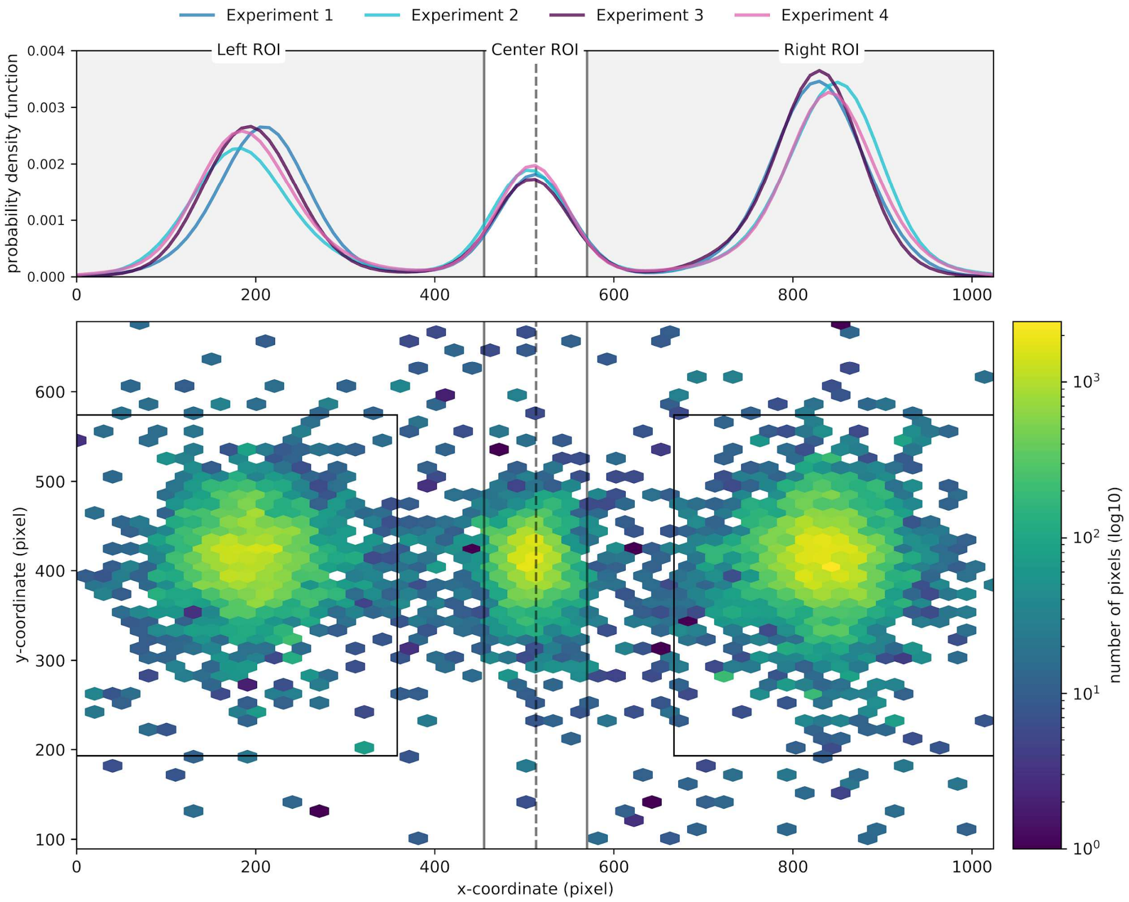

Figure S1. Map of visual fixations over the central and lateralized regions of interests. The superior panel depicts, in each experiment, the probability density function of the fixations of all valid trails of all infants over the x-axis, computed by applying the kernel density estimation technique. Grey areas indicated the x-pixel regions of the left and right regions of interest (ROI), and the white area indicates the x-pixel of central ROI. The inferior panel plots the 2D histogram of the screen coordinates over the x and y axis of each fixation, in logarithmic units according to the color code presented at the right side of the histogram. Black left and right frames show the areas where the two pictures or videos appeared simultaneously lateralized. The middle area indicates the region where the attractor was displayed before each trial onset.

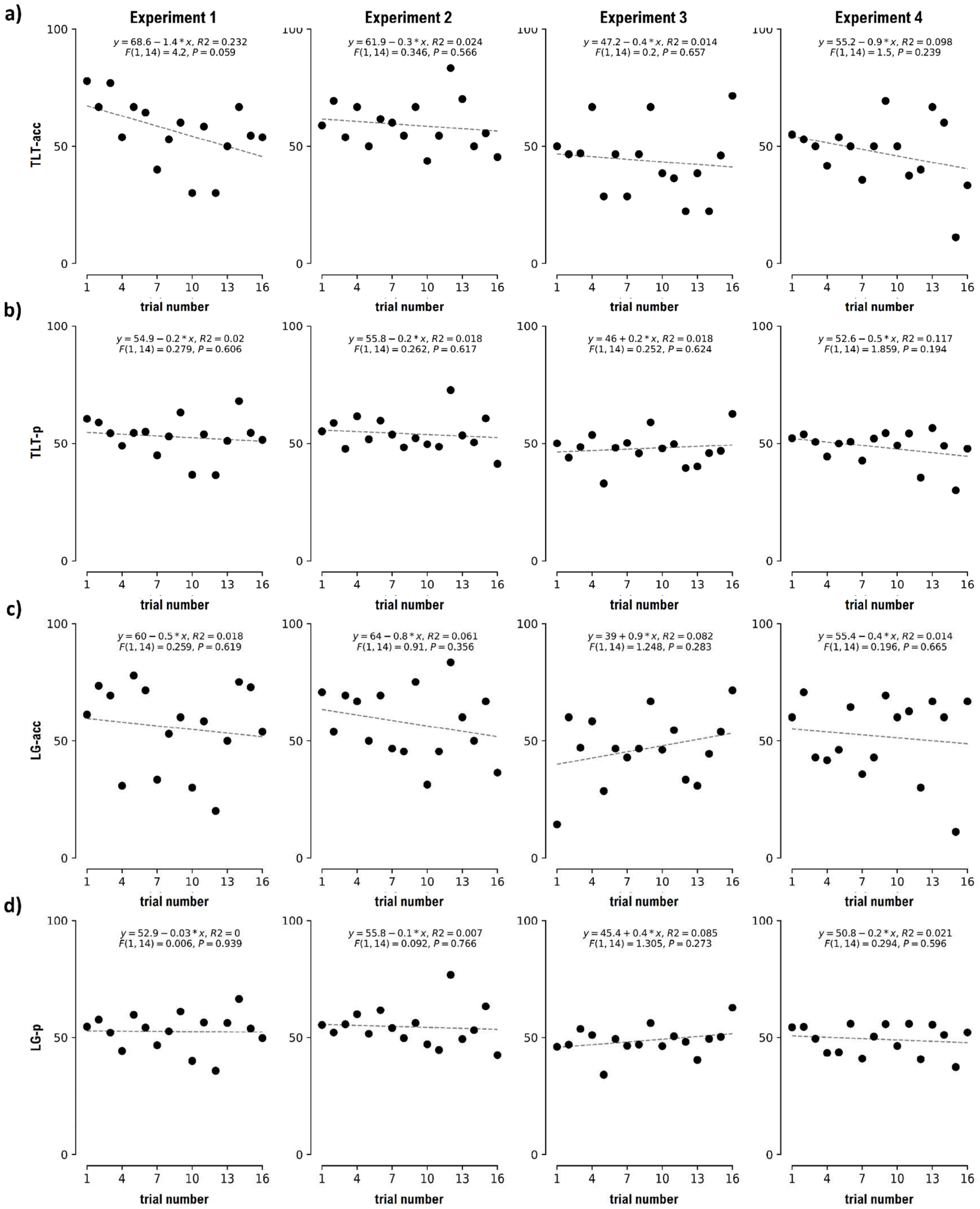

Figure S2. Infants did not learn during the test phase. Each graphic shows a simple linear regression (dotted line) for the trial number and TLT-acc in **a)**, TLT-p in **b)**, LF-acc in **c)** and LF-p in **d),** for the test phase in each experiment. The model, R2, and p-value of each linear model arepresented at the top of each graph. No significant correlations were found in any experiment (P > 0.059). Indeed, the trial number predictor's coefficient was mostly negative in all experiments, showing a tendency to decrease the performance as the task progressed. Therefore, we did not find data consistent with learning throughout the testing phase.

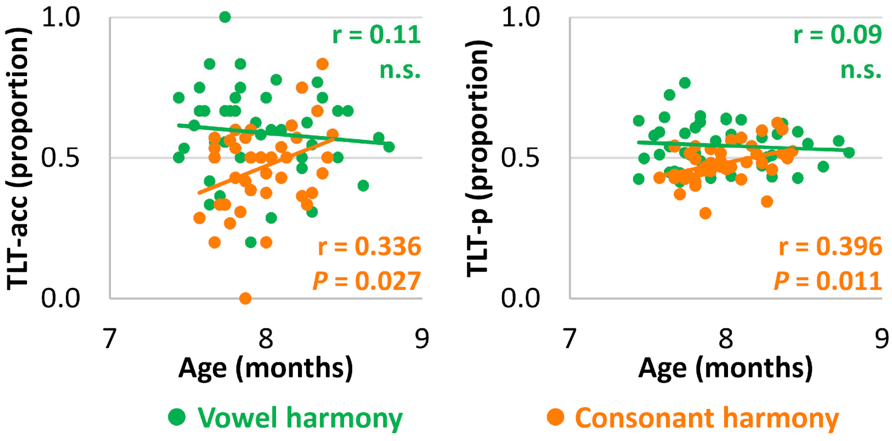

Figure S3. Infant's age correlates with performance. We plot the correlations between the infant age at the moment of the evaluation, and the performance of the infants evaluated in the two experiments providing vowel harmony (Experiment 1 and Experiment 2), and consonant harmony (Experiment 3 and Experiment 4). Although with performance at chance the infants exposed to audiovisual streams conveying consonant harmony exhibit a significant positive correlation.

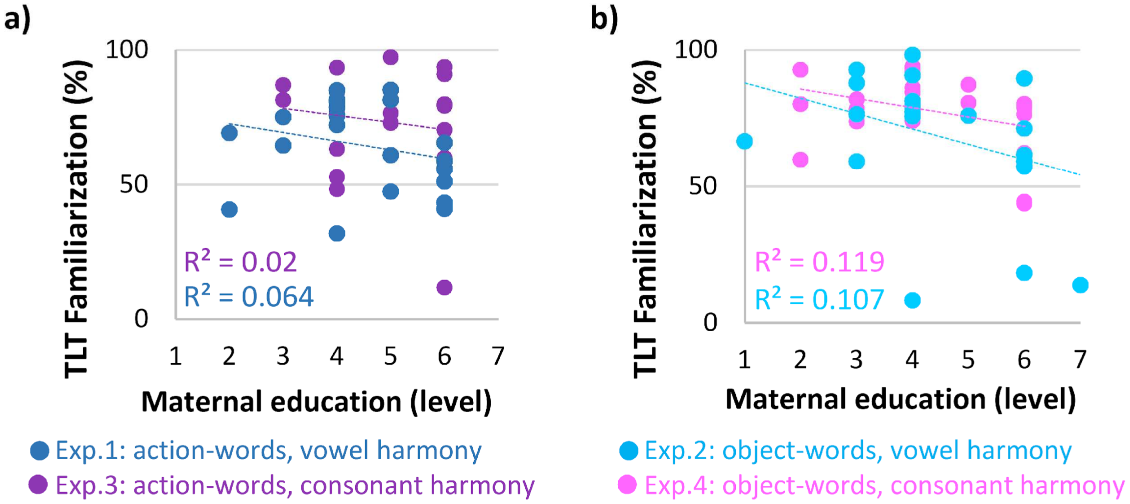

Figure S4. **The time exploring the screen during familiarization did not correlate with maternal education in each experiment**. In **a)** we plot the correlation of the total looking time (TLT) exploring the screen familiarization against the maternal education for Experiment 1 (in blue) and Experiment 3 (in purple), which measured the learning of word-action associations when action-words were cued by vowel and consonant harmony, respectively. In **b)** we show the correlation of the TLT during familiarization against the maternal education for Experiment 2 (in cyan) and Experiment 4 (in pink), which evaluated the learning of word-object associations, when object-words contained vowel and consonant harmony, respectively. R^2 indicated for each distribution.

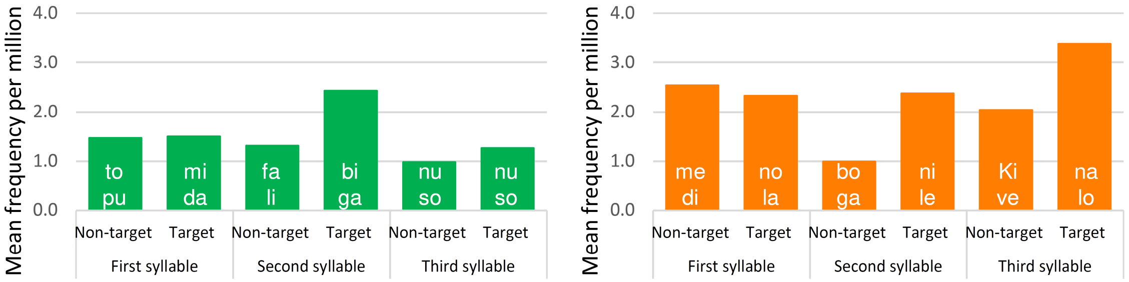

Figure S5. Frequency of the CV bigrams in Spanish. The mean frequency per million of the CV bigram of the target and non-target trisyllables was similarly distributed in experiments 1 and 2, providing vowel harmony cues, and in experiments 3 and 4, providing consonant harmony cues. The graphs show the histograms of the mean frequency per million (y-axis) for the syllables of target and non-target trisyllables, occurring at the first, second, and third syllables of the trisyllables, in Spanish(x-axis) computed in EsPal database (Douchon et al., 2013). The texts in each bar indicate the CV bigrams comprised in the computation of the mean frequency.

Supplementary Movies.

Brief examples of videos of the familiarization and testing phase used in all the studies. All videos last 27 s each. The first 7 s illustrate the familiarization and then we show 2 examples of the test trials. The words displayed syllable by syllable in the inferior part of each video are written just to clarify the synchrony of then audiovisual presentation, they did not appear in the original videos presented to infants. The audio codec is ACC and video codec is H.264. Videos are in MP4 format.

Movie S1. Familiarization and testing phase in Experiment 1. Action-words + vowel harmony.

Movie S2. Familiarization and testing phase in Experiment 2. Object-words + vowel harmony.

Movie S3. Familiarization and testing phase in Experiment 3. Action-words + consonant harmony.

Movie S4. Familiarization and testing phase in Experiment 4. Object-words + consonant harmony.

References

Duchon, A., Perea, M., Sebastián-Gallés, N. *et al.* EsPal: One-stop shopping for Spanish word properties. *Behav Res* 45, 1246–1258 (2013). https://doi.org/10.3758/s13428-013-0326-1
